# On the Morphology of the SDS Film on the Surface of Borosilicate Glass

**DOI:** 10.3390/ma10050555

**Published:** 2017-05-19

**Authors:** Zih-Yao Shen, Maw-Tien Lee

**Affiliations:** Department of Applied Chemistry, National Chia Yi University, Chiayi City 60004, Taiwan; s0982754@alumni.ncyu.edu.tw

**Keywords:** surfactant adsorption film, atomic force microscopy, sodium dodecyl sulfate

## Abstract

Surfactant films on solid surfaces have attracted much attention because of their scientific interest and applications, such as surface treatment agent, or for micro- or nano-scale templates for microfluidic devices. In this study, anionic surfactant sodium dodecyl sulfate (SDS) solutions with various charged inorganic salts was spread on a glass substrate and dried to form an SDS thin film. Atomic force microscopy (AFM) was employed to observe the micro-structure of the SDS thin film. The effects of inorganic salts on the morphology of the SDS film were observed and discussed. The results of experiments demonstrated that pure SDS film formed patterns of long, parallel, highly-ordered stripes. The existence of the inorganic salt disturbed the structure of the SDS film due to the interaction between the cationic ion and the anionic head groups of SDS. The divalent ion has greater electrostatic interaction with anionic head groups than that of the monovalent ion, and causes a gross change in the morphology of the SDS film. The height of the SDS bilayer measured was consistent with the theoretical value, and the addition of the large-sized monovalent ion would lead to lowering the height of the adsorbed structures.

## 1. Introduction

Surfactants are widely used as chemical ingredients in cleaning agents, emulsifiers, pigments, inks, anti-foaming agents, etc. When the surfactant solution is dribbled on a solid substrate, the solvent will evaporate, and the remaining particles will form various patterns on the surface. The morphology of the surfactant on the solid substrate depends on many factors, such as (i) the hydrophilic/hydrophobic property of the surface; (ii) the concentration of the surfactant; (iii) ionic functional groups of the surfactant and the surface charge; and (iv) counter-ions [1,2]. Due to scientific interest and promising applications (e.g., surface treatment agent [3,4] or micro- or nano-scale templates for microfluidic devices [5,6,7,8]), the design of the assembly structure has attracted much attention. Over the few past decades, many works have studied the morphology and the structure of ionic surfactants on the various substrates, including graphite, mica, silica, and metal [9,10,11,12,13,14,15,16,17,18,19,20,21,22,23,24,25,26,27]. The results of previous studies demonstrated that ionic surfactants could form flat sheets or semi-cylindrical structures on hydrophobic substrates via the van der Waals force, whereas the ionic surfactants would aggregate into the shape of a bilayer, cylinder, or sphere on the hydrophilic substrate through electrostatic interaction. 

Many researchers are interested in the effect of the ionic strength or the counter-ion on the organization of ionic surfactant molecules. Some studies have shown that the salts had an impact on the morphology of the ionic surfactant on the various surfaces when the ionic surfactant and the surface contained opposite charges [28]. Ducker et al. studied the influence of monovalent electrolytes on cetyltrimethylammonium bromide (CTAB) aggregates on mica [29]. The CTAB formed a flat sheet structure on the surface. With the addition of HBr and KBr to the system, the structure of the aggregate would transform into a cylindrical structure. Metal halide (CsBr, CsCl, KCl, LiCl, LiBr) salts could induce the transformation of the aggregate of CTAC on mica from bilayers to cylindrical micelles to globular micelles with a variation in concentration [30]. According to these studies, mica can bind cations because of its negative lattice sites. In the presence of salts, excess cations compete with positively-charged head groups of the surfactant to occupy the lattice sites and reduce the density of the surfactant on the surface. Thus, the shapes of the surfactant aggregates are transformed. 

The effect of salt on the morphology of the ionic surfactant adsorbed on the low-charge surface was studied. Ducker et al. used atomic force microscopy (AFM) to study the influence of NaCl on the aggregated structure of SDS on the graphite–solution interface [31]. The addition of NaCl to the system did not change the morphology of adsorbed surfactant, but it reduced the repulsive interactions between the negatively-charged head groups and resulted in a lower space between hemicylinders. The same result was evidenced by the study of molecular dynamics simulation [32]. For the divalent ions (Mg^2+^, Mn^2+^, and Ca^2+^), the adsorbed structures are very similar to the results observed in the presence of only monovalent counter ions. The high electric charge would enhance the adsorption density and did not change the shape of the aggregation conspicuously [33]. From the results of these studies, the hydrophobic tail groups of the ionic surfactant adsorbed on graphite by the van der Waals force and the addition of salts in SDS solutions does not greatly change the structures of adsorbed aggregate on graphite. 

As discussed previously, studies mainly discussed the effect of salts on the morphology or the transformation of the structure of the surfactant with the opposite charge of the surface, with little concern for the stacking of the surfactant and the effect of salts on the surfactant self-assembly on the surface with the same charge of the surfactant. In this study, the effect of salts on the stacking of the anionic surfactant on the negatively-charged surface was observed and discussed. Thin films of the anionic surfactant SDS with various charged inorganic salts were prepared on borosilicate glass, which is a hydrophilic and negatively-charged surface. AFM was employed to observe the structure of these films. The effects of various salts on the morphology of the SDS film are discussed.

## 2. Results

The morphology of the thin films are presented in three types of AFM image, which include the error signal image, the height image, and the 3D reconstruction. The height image displays the height of the surface with various colors. The 3D height image is useful for determining three-dimensional patterns of molecule accumulation.

### 2.1. Structure of the SDS Aggregate

**Figure 1:**
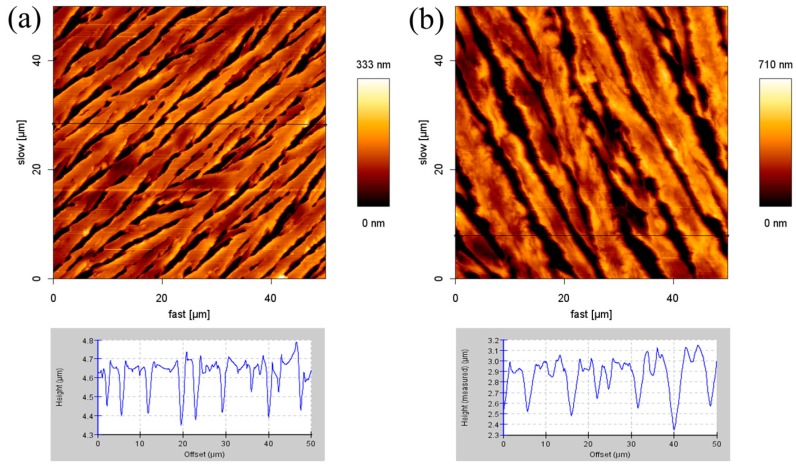
Atomic force microscopy (AFM) images (50 μm × 50 μm) and cross-section height profiles at: (**a**) 2 mM; and (**b**) 8.1 mM.

Figure 1a,b show the AFM surface height images and cross-section profiles of the morphology of SDS aggregates on the glass slide. Whether the concentration of SDS is 2 mM or 8.1 mM, the structure of the SDS molecules appear as linear and parallel stripes. The cross-section height profiles are along the black line in the AFM height image. This provides the height variation over the SDS aggregate structure. For the 2 mM SDS concentration, the adsorbed structures of 0.15–0.35 μm height and 3–8 μm width were observed. At the concentration of 8.1 mM (the critical micelle concentration (CMC) of the SDS solution), the adsorbed structures feature similar results. The height and the width of the structures are about 0.4–0.8 μm and 5–8 μm, respectively. The maximum height in these two samples are approximately 333 nm and 710 nm, respectively. These two images show that every stripe forms a flat layer and the distance between the stripes is about 2 μm. 

**Figure 2:**
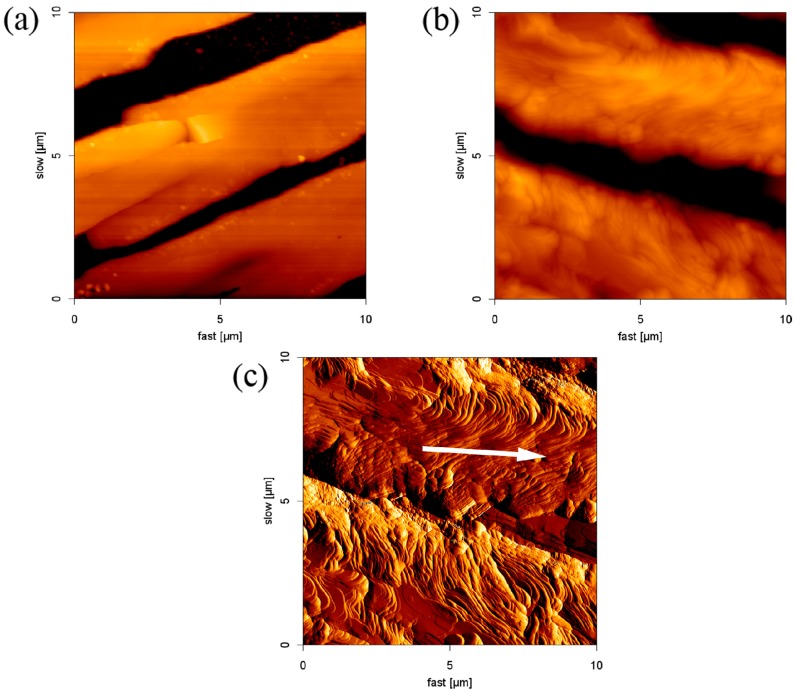
AFM images (10 μm × 10 μm) (**a**) at 2 mM and (**b**) at 8.1 mM; and (**c**) AFM error signal image from (b).

Figure 1a,b are enlarged and shown in Figure 2a,b, respectively. The edge of the parallel structure is smooth. Figure 2c is an AFM error signal image from Figure 2b. It is clear that SDS molecules self-assembled to form an ordered structure on borosilicate glass through layer-by-layer deposition. We also observed that the formation of the layer had an orientation outwards (indicated by the white arrow in Figure 2c). This phenomenon was assigned to the outward capillary flow during evaporation [34]. On the basis of the results, it is concluded that the height of the adsorbed structures are affected by the surfactant concentration, but the morphology and the aggregate structure do not change.

### 2.2. The Effect of the Cations on the Structure

**Figure 3:**
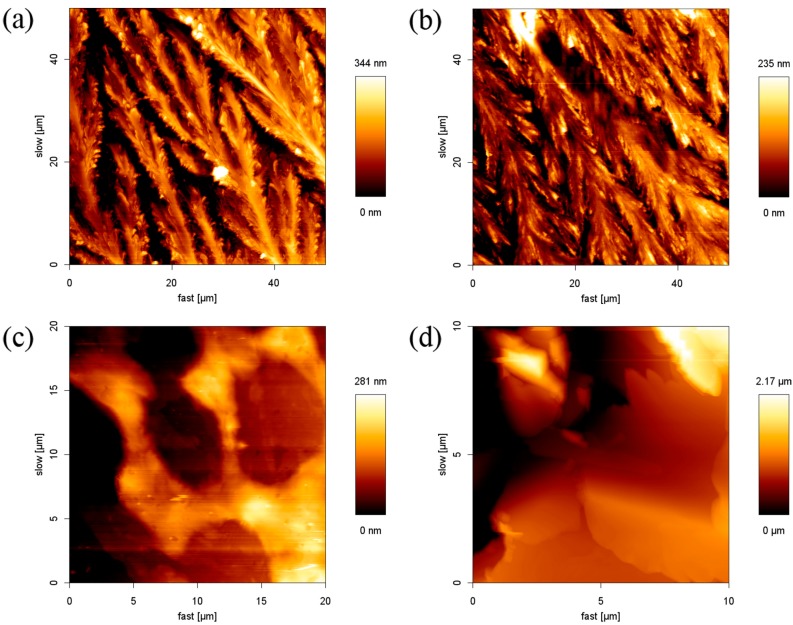
AFM images of SDS-inorganic salt aggregates on the surface of the glass: (**a**) SDS-NaCl; (**b**) SDS-KCl; (**c**) SDS-MgCl_2_; and (**d**) SDS-CaCl_2_.

For further understanding of the influence of the cations on the morphology of the SDS film, we prepared 8.1 mM SDS solutions with 8.1 mM alkali chloride (Na^+^, K^+^) or alkaline earth chloride (Mg^2+^, Ca^2+^) to form a thin film. Figure 3a,b show the results of the SDS-monovalent ion films. When SDS coexisted with monovalent ions, the transformation from the linear stripe to the rice plant shape was obtained. The SDS molecules in the flat layer structures stretched out, and the space between stripes decreased. The maximum height of the structure in SDS-NaCl and SDS-KCl are 344 nm and 235 nm, which are lower than that of the SDS film (710 nm). When SDS was mixed with the divalent cation, SDS molecules assembled in a disorderly manner and no regular structure was observed, as shown in Figure 3c,d. The results demonstrated that the existence of the inorganic salt would interfere with the structure of the SDS film. With enough electrolytes in the system, the counter-ions dominate in the Stern and diffuse layers, and hence reduce the repulsive interaction (increasing the attractive interaction) between DS^-^ molecules, so the counter-ions are more susceptible than co-ions. It is well-known that the valence and the concentration of counter-ions play an important role in the stability of the colloid system. In our case, all concentrations of SDS and electrolyte solutions were constant at 8.1 mM. Thus, the principal reason for the influence was ascribed to the valence of the counter ions. The phenomenon that SDS molecules aggregate intensely with the addition of multivalent ions can be formalized in the Schultz–Hardy rule for the critical coagulation concentration, as given in Equation (1):

(1) $$C.C.C.\;\propto \;\frac{\;1}{{z\;}^{6}}$$

where the critical coagulation concentration (C. C. C.) is defined as the minimum concentration of colloidal particles for coagulation and z is the valence of the counter-ions [35,36]. For z = 1 and 2, the ratio of the CCCs value are thus 1 and 0.0156. This means that colloid particles aggregate with each other at a lower concentration of higher-valence counter-ions. In other words, at the same concentration of electrolytes, high-valence counter-ions could result in a greater coagulation behavior. The divalent ion has much more electrostatic interaction with anionic head groups than that of the monovalent ion, so it causes a significant impact on changing the morphology of the SDS film.

### 2.3. The Effect of Anions on the Structure

**Figure 4:**
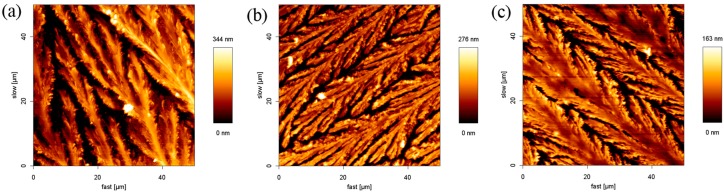
AFM images of SDS-inorganic salt aggregates on the surface of the glass: (**a**) SDS-NaCl; (**b**) SDS-NaI; and (**c**) SDS-NaNO_2_.

From the above experiments, the regular stripe structure of SDS was affected slightly by the addition of Na^+^ or K^+^ ions. To further understand whether the SDS structure could be influenced by the anions, the thin films from 8.1 mM SDS in the presence of 8.1 mM sodium inorganic salt (NaCl, NaI, or NaNO_2_) solutions were prepared, as shown in Figure 4. It was found that the maximum height of the structure in SDS-NaCl, SDS-NaI, and SDS-NaNO_2_ is 338 nm, 276 nm, and 163 nm, respectively. They are much lower than the height of the pure SDS film. It is clear that SDS structures near the periphery of the flat layer stretched out and a branched pattern was obtained. The results are very similar to that observed in the presence of NaCl and KCl.

### 2.4. Formation of SDS Multilayers

**Figure 5:**
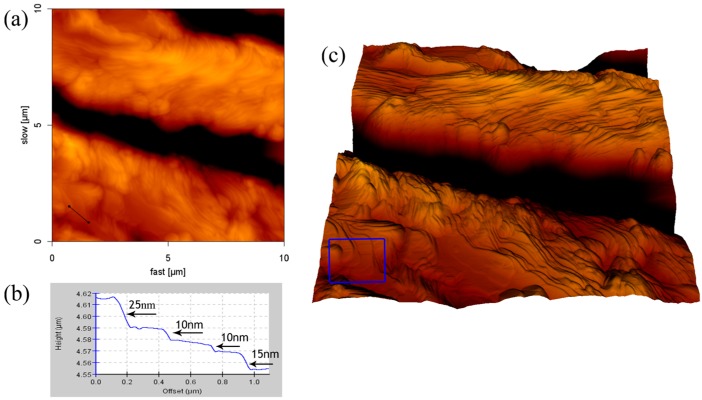
AFM result of SDS aggregates of the surface of the glass: (**a**) height image; (**b**) cross-section height profiles along the line in (a); and (**c**) 3D reconstruction image.

**Figure 6:**
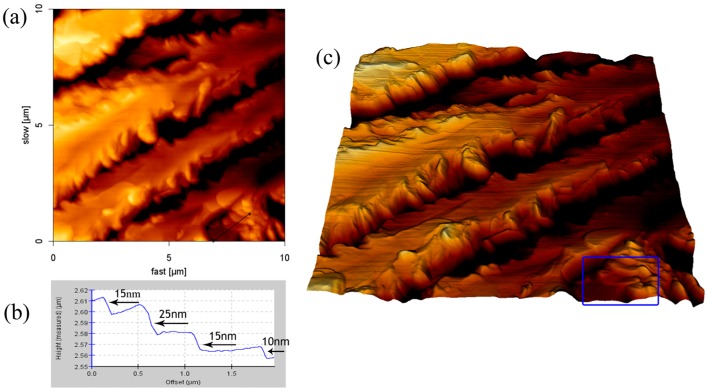
AFM result of SDS-NaCl aggregates of the surface of the glass: (**a**) height image; (**b**) cross-section height profiles along the line in (a); and (**c**) 3D reconstruction image.

We can observe the stacking formation of the aggregation by using AFM cross-section profile and 3D reconstruction image. The film structure formed by using the 8.1 mM SDS solution was shown in Figure 5. It can clearly be seen in the 3D reconstruction image (Figure 5c) that the SDS adsorption on borosilicate glass was stacked in layers. The cross-section of an indentation site in the AFM height image (Figure 5b) shows that the thicknesses of the SDS layers are approximately 10, 15, or 25 nm. The structure of the SDS-NaCl film is represented in Figure 6. The 3D reconstruction image—which is on the periphery of the aggregation structure—shows that the SDS molecules in the flat layer structures extended laterally and had the lower space between structures. The cross-section profile reveals that the thicknesses of the aggregated layers are approximately 10, 15, or 25 nm, which is similar to the result of SDS film.

**Figure 7:**
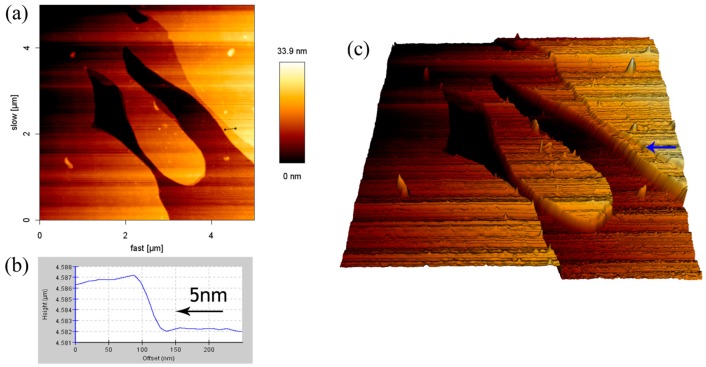
AFM result of SDS-CsCl aggregates of the surface of the glass: (**a**) height image; (**b**) cross-section height profiles along the line in (a); and (**c**) 3D reconstruction image.

It is known from the previous results that the low concentration of the SDS solution would cause a lowering of the height of the adsorbed structure from 0.7 μm to 0.2 μm. Without changing the concentration of SDS, the addition of the monovalent ions could also cause a similar result. It is interesting to note the effect of the large-volume cations on the height of the SDS adsorbed film. The film structure formed by using the solution with 8.1 mM SDS-CsCl was recorded by AFM, as shown in Figure 7. The branched pattern was observed and the maximum height of the film decreased to 33.9 nm, which is far lower than that of the other SDS-monovalent ion system. The 3D reconstruction image (Figure 7c) shows that the SDS structure was in the form of lamellae. It is clear that the SDS adsorption on borosilicate glass was stacked in layers. The cross-section of an indentation site in the Figure 7a depicts that the thickness of the SDS layers is approximately 5 nm. According to previous literature, the hydrophobic part of one SDS bilayer at low water content (1:8 water:SDS) is about 4 nm, and each sulfate group has 0.6 nm length [22,37,38]. Hence, the result corresponds to the height of one surfactant bilayer.

## 3. Discussion

**Figure 8:**
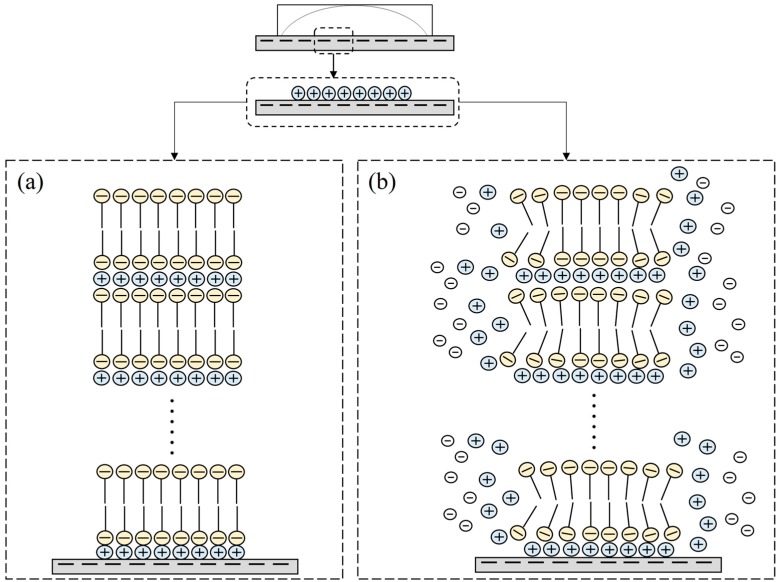
Schematic representation of possible mechanisms for surfactant adsorption on a glass slide: (**a**) SDS; and (**b**) SDS-monovalent ions.

In previous literature, the SDS formed hemicylindrical structure on the graphite surface since the hydrophobic substrates interact with the carbon chains through van der Waals forces [19,31]. According to the AFM results mentioned above, we observed the stacking formation and the morphology of the aggregates, which is similar to the aggregation of SDS on the mica and ZnSe surface [22,23]. These observations provide some information to postulate the mechanism of the adsorbed process as represented in Figure 8. It is well-known that borosilicate glass is a negatively-charged surface with hydrophilic properties, and the DS^-^ has a negatively-charged head group. Figure 8a gives the possible mechanism for the formation of the SDS film. When the SDS solution is dropped on the glass surface, the negatively-charged sites of the borosilicate glass binds the cations through attractive electrostatic interaction [30]. The negative DS^-^ head group adsorbs on the Na^+^ ion layer, and the hydrophobic carbon chain of the SDS is upward. Then, the hydrophobic carbon chains of the SDS link together by the van der Waals force to form a deposited bilayer. In the drying process, the two parallel and negatively-charged surfaces are forced to approach mutually and create a thin layer between the two surfaces. This thin layer has the potential for counter ions to compensate the charge repulsion. Thus, Na^+^ ions penetrate into the negative interface between the DS^-^ head groups to compensate for the charge density and result in a bilayer [22]. Finally, a lamellar structure of stacking bilayers forms. 

Ducker et al.’s study indicates that ionic strength has an influence on aggregate space [31]. The space between aggregates can be controlled by Coulombic interactions. When the salt concentration increased (high ionic strength), excess cations reduced repulsive interactions between headgroups and decreased the space between stripes. SDS molecules were attracted by the excess monovalent cations and extended laterally instead of stacking upward, which caused the stripe shape of SDS to change to the rice plant/leafy shapes and the reduction of the stack thickness, as shown in Figure 8b. Thus, the height of the structure is much lower than that of the pure SDS film. Since the large size of cesium ions results in a lower charge density, the electrostatic interaction force between these ions and the substrate is poor. Thus, the ions prefer to spread with the solvent to flow in preference of binding onto the liquid–solid interface. The SDS molecules are also susceptible to movement, giving rise to a wider range of diffusion. The mutual attractive force between SDS molecules for stacking is low in this case. Hence, we still obtain the SDS bilayer by adding the larger ions without decreasing the SDS concentration.

## 4. Materials and Methods 

All water in the experiment was pre-treated by a Milli-Q system and had a conductivity of 18 MO cm^−1^. Sodium dodecyl sulfate was purchased from Sigma (purity, 92.5–100.5%). NaCl (J.T. Baker, 99.7%), KCl (Choneye pure chemicals, 98%), CsCl (Merk, 99.5%), CaCl_2_·2H_2_O (Hayashi Japan, 99.7%), MgCl_2_ (Honeywell Riedel-de Haen chemicals, 99%), NaI (Choneye pure chemicals, 99%), and NaNO_2_ (Showa chemical, 98.5%) were used as the electrolyte additives. SDS was prepared at 2 mM and 8.1 mM. SDS-inorganic salt aqueous solutions were prepared at 8.1 mM. 

The borosilicate glass slide was cleaned with 75% ethanol to remove possible organic impurities and then rinsed with ultrapure water from the Milli-Q system before use. A drop of solution with a volume of 15 μL was transferred to the glass surface with a pipette. The borosilicate glass with the solution drop was kept at 25 °C and a humidity of 60% for 24 h. The water was evaporated, and the surfactant film formed. All films were imaged by atomic force microscopy (JPK, Axiovert 200, Berlin, Germany) in intermittent contact mode. The AFM probes were Tap 150Al-G silicon probes, which were purchased from Budget Sensors. 

## 5. Conclusions

In this study, the self-aggregation of sodium dodecyl sulfate with inorganic salts on the surface of the borosilicate glass was investigated by AFM. The results of the experiments demonstrated that pure SDS film formed patterns of long, parallel, highly-ordered stripes. The existence of inorganic salt disturbed the structure of the SDS film due to the interaction between cationic ions and the anionic head groups of SDS. The branch pattern was obtained by adding the monovalent ion. The divalent ion has greater electrostatic interaction with anionic head groups than that of the monovalent ion, and has a greater effect in changing the morphology of the SDS film.

The addition of a large-sized monovalent ion led to a lowering of the height of the adsorbed structures without adjusting the SDS concentration. By using CsCl, a lamellar structure was obtained. It was evidenced that SDS molecules were self-assembled on borosilicate glass through layer-by-layer deposition. The height of the lamella (about 5 nm) was in agreement with the thickness of an SDS bilayer that was estimated from theories in the literature.
